# Effects of 12-Week *Bacopa monnieri* Consumption on Attention, Cognitive Processing, Working Memory, and Functions of Both Cholinergic and Monoaminergic Systems in Healthy Elderly Volunteers

**DOI:** 10.1155/2012/606424

**Published:** 2012-12-18

**Authors:** Tatimah Peth-Nui, Jintanaporn Wattanathorn, Supaporn Muchimapura, Terdthai Tong-Un, Nawanant Piyavhatkul, Poonsri Rangseekajee, Kornkanok Ingkaninan, Sakchai Vittaya-areekul

**Affiliations:** ^1^Neuroscience Program, Department of Physiology and Graduate School, Faculty of Medicine, Khon Kaen University, Khon Kaen 40002, Thailand; ^2^Department of Physiology, Faculty of Medicine, Khon Kaen University, Khon Kaen 40002, Thailand; ^3^Integrative Complimentary Alternative Medicine Research and Development Group, Khon Kaen University, Khon Kaen 40002, Thailand; ^4^Department of Psychiatry, Faculty of Medicine, Khon Kaen University, Khon Kaen 40002, Thailand; ^5^Department of Pharmaceutical Chemistry and Pharmacognosy, Faculty of Pharmaceutical Sciences, Naresuan University, Phitsanulok 65000, Thailand

## Abstract

At present, the scientific evidence concerning the effect of *Bacopa monnieri* on brain activity together with working memory is less available. Therefore, we aimed to determine the effect of *B. monnieri* on attention, cognitive processing, working memory, and cholinergic and monoaminergic functions in healthy elderly. A randomized double-blind placebo-controlled design was utilized. Sixty healthy elderly subjects (mean age 62.62 years; SD 6.46), consisting of 23 males and 37 females, received either a standardized extract of *B. monnieri* (300 and 600 mg) or placebo once daily for 12 weeks. The cholinergic and monoaminergic systems functions were determined using AChE and MAO activities. Working memory was assessed using percent accuracy and reaction time of various memory tests as indices, whereas attention and cognitive processing were assessed using latencies and amplitude of N100 and P300 components of event-related potential. All assessments were performed before treatment, every four weeks throughout study period, and at four weeks after the cessation of intervention. *B. monnieri*-treated group showed improved working memory together with a decrease in both N100 and P300 latencies. The suppression of plasma AChE activity was also observed. These results suggest that *B. monnieri* can improve attention, cognitive processing, and working memory partly via the suppression of AChE activity.

## 1. Introduction

It is well established that aging is associated with a gradual impairment of cognitive function [1]. Age-related cognitive ability decline varies considerably across individuals and across cognitive domains. Various cognitive domains show different susceptible to aging. The basic cognitive functions most affected by age are speed of processing, memory, spatial ability, and reasoning [2]. Since the cognitive function is a key success factor in life, the strategy to sustain or prolong this function is one of the ultimate goals in care for the elderly. Therefore, the development of cognitive enhancers has been focused on in research.

Currently, the effects of aging on cognitive function have become a prominent area of research. An understanding of time-course perception, attention-related, and cognitive processing can be provided by a series of well-characterized event-related potential (ERP) components [3, 4]. It has been proposed that age-related negative deflection, or N100, which is observed between 80 and 120 milliseconds after stimuli, reflects attention process. The amplitude of N100 component is modulated by selective attention. Auditory N100 is larger for attended than for unattended stimuli [5]. Besides the N100 component, P300, or positive deflection with latency of 250–500 milliseconds after stimuli, is also regarded as an important marker reflecting attention and memory operations [6, 7]. The relationship between topographical location of electrode and event-related potential (ERP) components, especially N100 and P300, shows good reliability at midline location (via Fz, Pz, and Cz) of more than 0.7. Both amplitudes and latencies of N100 and P300 components are influenced by aging. The latencies of P300 and N100 components were increased, whereas the amplitudes of these two components were decreased [8–12].

*Bacopa monnieri* Linn or Brahmi, a plant in the family Scrophulariaceae, has been used in the Ayurvedic system of medicine for centuries. It has been claimed as a nerve tonic and extensively used for treatment of various neurological and neuropsychiatric diseases. Accumulative lines of evidence have demonstrated that *B. monnieri* extract could enhance memory in animal studies [13–15]. In addition, it has been demonstrated that consumption of *B. monnieri* at a dose of 300 mg per day can enhance logical memory, paired associated learning, and mental control in age-associated memory impairment (AAMI) subjects without serious adverse effects. However, “flu-like symptoms” and “digestive problems” were also observed [16]. A systematic review of studies also shows that consumption of *B. monnieri* at doses of 300–450 mg/day can enhance memory recall [17–22]. To date, little scientific evidence concerning the effect of various doses of *B. monnieri* on brain activities and neurotransmitters changes in human volunteers is available. Based on the data obtained from animal studies of our groups, it was found that *B. monnieri* at doses of 20–80 mg/kg BW could enhance neuron density in the hippocampus, the area contributing an important role to learning and memory. The optimum benefit was observed at a dose of 40 mg/kg BW [14]. Therefore, we calculated this dose to a human equivalent dose (HED) with a safety factor of 4 because this plant is quite safe. LD50 via oral route was approximately 17 g/kg BW [22]. To the best of our knowledge, no scientific documentation concerning the effect of *B. monnieri* at a dosage range between 300 and 600 mg per day on brain activities and neurotransmitters changes exists, hence the need for this study. Since *B. monnieri* has previously been shown to possess a cognitive enhancing effect, we hypothesized that *B. monnieri* might alter the function of the cholinergic system giving rise to enhanced attention and cognitive processing capability and finally leading to enhanced working memory. To test this, the present study was set up to determine the effect of *B. monnieri* on working memory, N100 and P300, together with the alteration of acetylcholine and the monoamine neurotransmitter in attention and cognitive processing.

## 2. Materials and Methods

### 2.1. Participants

A total of 60 healthy elderly volunteers (mean age 62.62 ± 6.46 years) were recruited to participate in this study. All participants gave their written, informed consent. The study protocol was approved by the Khon Kaen University Ethical Committee on Human Research. Prior to participation, each volunteer was requested to complete a medical health questionnaire. Then, all the subjects underwent extensive medical evaluation in order to ascertain subject suitability for entering the phase of clinical trial. Additionally, they were selected to be free of any herbal or prescribed medication that interfered with the function of the nervous system. Habitual smokers consuming more than 10 cigarettes/day and any subjects who would have had difficulty in abstaining from smoking during the study duration were excluded from this study. All participants had abstained from caffeine containing products throughout each study day and alcohol for a minimum of 12 h prior to the test session. They were divided into three groups; placebo, *B. monnieri* 300, or *B. monnieri* 600 mg/day. No one dropped out from the study. Moreover, no significant differences in mean age, education, and body mass index among groups were observed.

### 2.2. *Bacopa monnieri* Preparation

**Figure 1:**
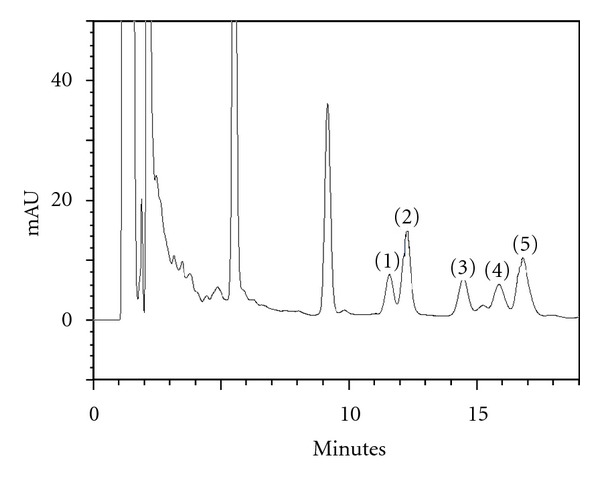
HPLC-chromatograms of *B. monnieri* extract (2 mg/mL) containing 5% (w/w) of total saponins comprising a mixture of (1) Bacoside A_3_, (2) Bacopaside II, (3) Bacopaside X, (4) Bacopasaponin C, and (5) Bacopaside I.

A standardized tablet comprising either 300 or 600 mg of a proprietary dried *B. monnieri* extract was prepared by the Faculty of Pharmaceutical Sciences at Naresuan University in Phitsanulok and authenticated by Associate Professor Wongsatit Chuakul of the Faculty of Pharmacy at Mahidol University, Thailand. The voucher specimen (Phrompittayarat 001) was kept at the Pharmaceutical Botany Mahidol Herbarium of Mahidol University, Thailand [23]. In brief, the aerial part of *B. monnieri* was collected, cut into small pieces and dried in a hot-air oven at 50°C and then crushed. This dried powder was percolated for 8 h with 95% ethanol (1 g in 6 mL) three times and then filtered. The filtrate was evaporated under reduced pressure. The percent yield obtained was 10%. The total saponin content was determined using HPLC 5% (w/w) of the crude ethanol extract and was comprised of a mixture of bacoside A_3_, bacopaside II, bacopasaponin X, bacopasaponin C, and bacopaside I (Figure 1). Standardization and conformity of the extract was conducted by combination with pharmaceutical grade excipients with in-process controls during manufacture and complete analytical control according to petit patent number 0703000101 (2009). Each tablet contained approximately 300 and 600 mg of the crude extract of *B. monnieri*. Placebo tablets were manufactured using the same pharmaceutical excipients and replicated the active ones in appearance, odor, and texture. Packaging and randomization was performed, and then the trial was conducted by the study coordinator of the Integrative Complimentary and Alternative Medicine Research and Development Group of Khon Kaen University. 

### 2.3. Procedures and Treatments

This study was a pilot study conducted as a 12-week double-blind and placebo-controlled randomized trial. A random list of numbers was determined by a computer-generated series with the proper sequence applied to container labels and supplied to participants in the order enrolled after being randomly assigned to the various treatment groups. Each participant received one tablet of placebo or a *B. monnieri* extract tablet containing either 300 or 600 mg once daily using randomized double-blind coding, that is, neither researchers nor the subjects knew which type of treatment the subjects received. The placebo and *B. monnieri* tablets had the same color, texture, size, and smell. All participants were assessed for baseline data about working memory, cognitive function, and the alteration of the activity of acetylcholinesterase (AChE) and monoamine oxidase (MAO). Then, they were assessed according to the above-mentioned parameters every 4 weeks throughout the 12 weeks of treatment and at four weeks after treatment. The code for study allocation was only broken when the last participant completed the entire followup. The staffs involved in the collection of the study's endpoints were instructed to follow a rigorous protocol and not to discuss any issues related to the use of medication. Reviews for compliance with medication and side effects were performed independently by the investigators, who were also blinded to group allocation. Subjects were asked to call the study center if they experienced any medical problems during the 90-day study period. At the end of the study, they were also asked about adverse events. Laboratory tests were drawn at baseline and at follow-up visits and compared to see whether any changes suggested adverse events.

### 2.4. Working Memory

Four domains of working memory comprising power of attention, continuity of attention, quality of memory, and speed of memory were assessed via computerized battery test as described elsewhere [24, 25]. In this study, test-retest reliability in each domain of working memory varied between 0.72 and 0.82 (power of attention = 0.75, continuity of attention = 0.78, quality of working memory = 0.82, and speed of memory = 0.72). A selection of computer-controlled tasks from the system was administered. Task presentation was performed via VGA color monitors, and all responses were recorded via two-button (YES/NO) response boxes. The entire selection of tasks presented took approximately 20 minutes. Tests were administered in the following order.

**Word Presentation**

Fifteen words, matched for frequency and concreteness, were presented in sequence on the monitor for the participant to remember. The stimulus duration was 1 s, as was the interstimulus interval.

**Picture Presentation**

A series of 20 photographic images was presented on the monitor at the rate of 1 every 3 s, with stimulus duration of 1 s, for the participant to remember.

**Simple Reaction Time**

The participant was instructed to press the “yes” response button as quickly as possible every time the word “yes” was presented on the monitor. Fifty stimuli were presented with an interstimulus interval that varied randomly between 1 and 3.5 s. Reaction times were recorded in milliseconds.

**Digit Vigilance Task**

A target digit was randomly selected and constantly displayed to the right of the monitor screen. A series of digits was presented in the center of the screen at the rate of 80 min^−1^, and the participant was required to press the “yes” button as quickly as possible every time the digit in the series matched the target digit. The task lasted 1 min and there were 15 stimulus-target matches. Task measures were accuracy (%), reaction time (milliseconds), and number of false alarms.

**Choice Reaction Time**

Either the word “no” or the word “yes” was presented on the monitor, and the participant was required to press the corresponding button as quickly as possible. There were 50 trials, in which the stimulus word was chosen randomly with equal probability, with a randomly varying interstimulus interval of between 1 and 3.5 s. Reaction time (millisecond) and accuracy (%) were recorded.

**Spatial Working Memory**

A pictorial representation of a house was presented on the screen with four of its nine windows lit. The participant was instructed to memorize the position of the illuminated windows. In 36 subsequent presentations of the house, one of the windows was illuminated and the participant decided whether or not this matched one of the lighted windows in the original presentation. The participant made their response by pressing the “yes” or “no” response button as quickly as possible. Mean reaction times were measured in milliseconds, and the accuracy of responses to both original and novel (distracter) stimuli was recorded as percentages that were used to derive a “percentage greater than chance performance” score.

**Numeric Working Memory**

Five digits were presented sequentially for the participant to hold in memory. This was followed by a series of 30 probe digits for each of which the participant decided whether or not it had been in the original series and pressed the “yes” or “no” response button as appropriate as quickly as possible. This was repeated two further times with different stimuli and probe digits. Mean reaction times were measured in milliseconds and the accuracy of responses to both original and novel (distracter) stimuli were recorded as percentages that were used to derive a “percentage greater than chance performance” score.

The data were presented as mean ± SD of 4 domains as follows: (1) power of attention, a measure of attention and psychomotor/information processing speed was derived by combining reaction times of three attentional tasks: simple reaction time, choice reaction time, and digit vigilance (units are summed milliseconds for the three tasks). (2) Continuity of attention, a measure of attention summing accuracy of attention, was derived by calculating the combined percentage accuracy across the choice reaction time and digit vigilance tasks. 100 percent accuracy across the two tasks would result in a maximum score of 200. (3) Quality of memory, a measure of working memory summing accuracy, was obtained by combining the percentage accuracy scores spatial working memory, numeric working memory, word recognition, and picture recognition. 100 percent accuracy across the four tasks would generate a maximum score of 400. (4) Speed of memory, a measure of complex information processing speed, was derived from summing reaction times of the numeric and spatial working memory, word and picture recognition tasks.

### 2.5. Event-Related Potential Assessment

An Ag-AgCl electrode was placed at Cz actively, and linked mastoids were used as reference for the electrodes. Impedances were maintained below 5 kΩ. Both N100 and P300 amplitude and latencies were elicited with a standard auditory oddball paradigm. Frequent and target tones were presented binaurally through headphones. Participants were instructed to listen for and count infrequent target tones (650 Hz, 60 dB, 200 ms), which occurred randomly amongst 82–90 frequent nontarget tones (1 kHz, 60 dB, 200 ms). Interstimulus intervals varied randomly between 1,250 and 3,000 ms. The latency range in which the N100 and P300 maximum amplitudes and latencies were determined was between 65–135 and 280–375 ms, respectively.

### 2.6. Determination of the Activity of Acetylcholinesterase (AChE) Activity

After 8 hours of fasting, venous blood was drawn from each subject and immediately prepared as plasma to determine the AChE and MAO activities. AChE activity was evaluated using amount of nitrobenzoate liberated via the reaction of thiocholine and dithiobis-nitrobenzoic acid as index [26]. The determination of nitrobenzoate was performed at 405 nm. 

### 2.7. Determination of Monoamine Oxidase (MAO) Activity

MAO activity was determined by microplate assay. The determination of MAO activity used in this study was based on the concept that in the presence of a suitable amine substrate, amine oxidase enzymes generate hydrogen peroxide, which then drives the peroxidase-dependent oxidation of 4-aminoantipyrine. A subsequent interaction with vanillic acid generates stoichiometric amounts of a red quinoneimine dye, the appearance of which is monitored at 498 nm.

### 2.8. Statistical Analysis

All data are expressed as mean ± SD. Repeated measures ANOVA for cognitive domains and ERPs data were conducted. This was followed at each time point with Tukey's HSD post hoc comparison. Statistical significance was regarded at *P* value < 0.05.

## 3. Results

### 3.1. Demographic Data of Subjects

The baseline demographic data of the subjects in all groups are shown in Table 1. No significant difference was observed in all parameters among the three groups.

### 3.2. Effect of *B. monnieri* on the Working Memory

**Figure 2:**
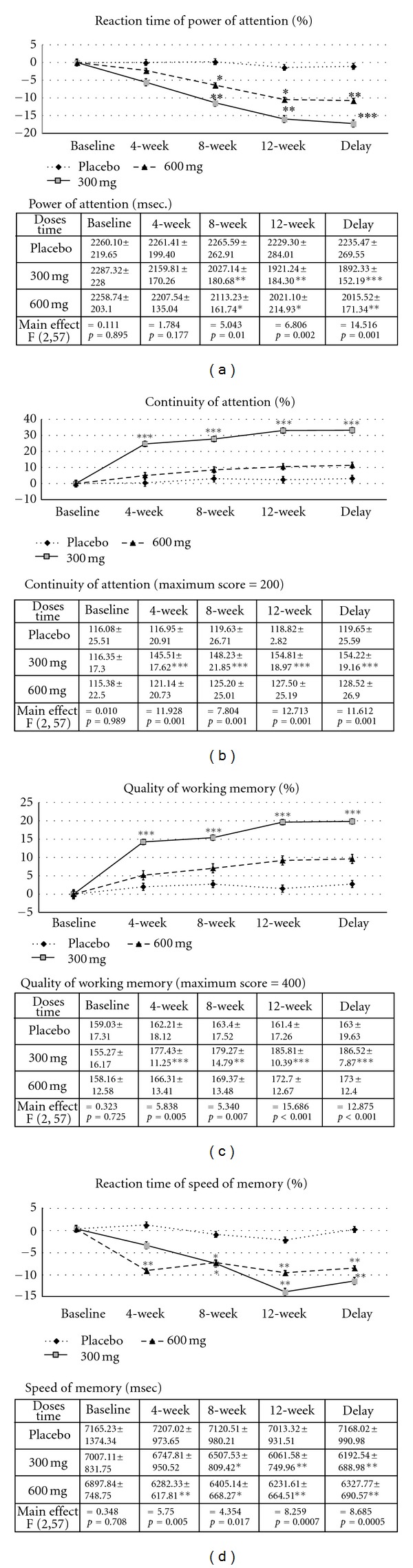
Effect of *B. monnieri* on percent change from baseline of (a) reaction time of power of attention, (b) continuity of attention, (c) quality of working memory, and (d) reaction time of speed of memory. * = *P* < 0.05, ** = *P* < 0.01, *** = *P* < 0.001 when compared to placebo-treated group.

The effects of *B. monnieri* on the 4 main domains of working memory are shown in Figure 2. After 4 weeks of administration of *B. monnieri*, the subjects who received *B. monnieri* at dose of 300 mg·day^−1^ showed significant enhancement in both the continuity of attention and the quality of memory (*P* value < .001; compared to placebo-treated group). These changes continued up to the 12th week of experiment and at the week 4 period after the cessation of *B. monnieri* consumption (*P* value < .001; compared to placebo-treated group). 

The subjects who consumed *B. monnieri* at a dose of 600 mg·day^−1^ showed a significant increase in the speed of memory (*P* value < .01; compared to placebo-treated group) at 4 weeks of treatment. When the intervention duration was further increased to 8 and 12 weeks, significant changes in this parameter were observed in subjects who consumed the extract both at doses of 300 and 600 mg·day^−1^ (*P* value < .05 and *P* value < .01; compared to placebo-treated group). Even at week 4 period after the cessation of *B. monnieri* consumption, these significant changes were still observed in both groups of *B. monnieri* treatment (*P* value < .01; compared to placebo-treated group). At 8-week consumption of *B. monnieri*, subjects who consumed *B. monnieri* at both doses demonstrated significantly enhanced power of attention (*P* value < .01 and .05, *P* value < .01 and .05, resp.; compared to placebo-treated group). Again, improved reaction time of power of attention was still observed in subjects who consumed *B. monnieri* at doses of 300 and 600 mg·day^−1^ at the 12-week intervention period and at 4 weeks after the cessation of *B. monnieri* consumption (*P* value < .01 and .05, *P* value < .001 and .01, resp.; compared to placebo-treated group).

### 3.3. Effect of *B. monnieri* on Event-Related Potential

The effects of *B. monnieri* on attention and cognitive processing were evaluated via N100 and P300 components of event-related potential, and results are shown in Table 2. No significant changes in both N100 and P300 were observed prior to the intervention. At the week 8 consumption period, subjects who consumed *B. monnieri* extract at a dose of 300 mg·day^−1^ demonstrated significantly decreased P300 latency (*P* value < .05; compared to placebo-treated group). This change was still observed at the week 12 consumption period (*P* value < .01; compared to placebo-treated group). The decreased N100 latency was also observed in subjects who consumed *B. monnieri* at both doses (*P* value < .001, .05, resp.; compared to placebo-treated group) at the week 12 intervention period. In addition, subjects who consumed *B. monnieri* extract at the dose of 600 mg·day^−1^ also showed decreased P300 latency (*P* value < .05; compared to placebo-treated group). Unfortunately, no significant changes were observed at 4 weeks after the cessation of extract consumption.

### 3.4. Effect of *B. monnieri* on the Activities of AChE and MAO

**Figure 3:**
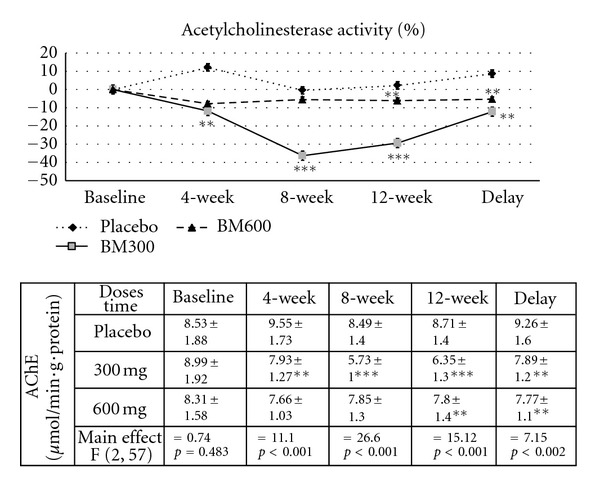
Effect of *B. monnieri* on percent change from baseline of Acetylcholinesterase activity throughout the study period. (*N* = 20/group) * = *P* < 0.05, ** = *P* < 0.01, *** = *P* < 0.001 when compared to placebo.

**Figure 4:**
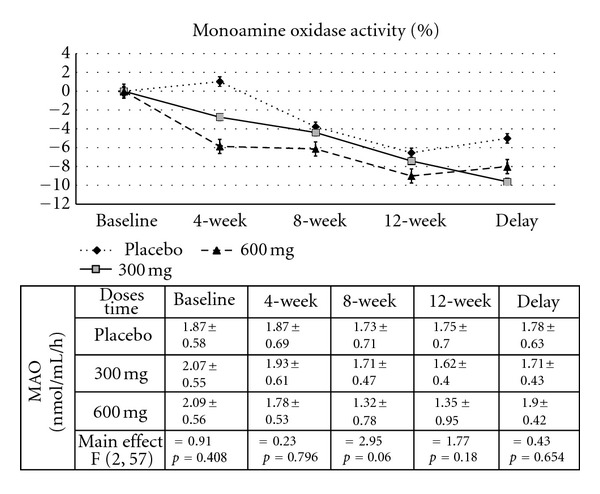
Effect of *B. monnieri* on percent change from baseline of Monoamine oxidase activity throughout the study period. (*N* = 20/group).

Since acetylcholine and monoamine transmitters play a crucial role in cognitive function especially during attention and cognitive processing, we also determined changes of both transmitters using the activities of both enzymes as indices, and results are shown in Figures 3 and 4. It was found that subjects who consumed the extract at the dose of 300 mg·day^−1^ showed a significant reduction of AChE activity at the week 4 consumption period (*P* value < .01; compared to placebo-treated group). The increased intervention period to 8 and 12 weeks also showed significant changes (*P* value < .001 all; compared to placebo-treated group). At the week 12 consumption period, the subject who consumed *B. monnieri* at the dose of 600 mg·day^−1^ also showed a significant reduction of AChE activity. The significant changes observed in subjects who consumed *B. monnieri* at the dose of 300 and 600 mg·day^−1^ were still observed at week 4 after the cessation of extract consumption (*P* value < .01 all; compared to placebo-treated group). However, no significant changes in MAO activity were found, as shown in Figure 4.

### 3.5. Adverse Effects

The current data has not shown serious adverse effects associated with *B. monnieri* use during the trial with the 300 mg and 600 mg dosages or the placebo. No changes on hematological and biochemical values indicating toxicity were observed. ECG recordings revealed no abnormalities and were within normal limits. No subjects drop outs of the study.

## 4. Discussion

The present data clearly demonstrates that *B. monnieri* especially at a dose of 300 mg·day^−1^ could improve power and speed of attention, continuity of attention, and quality and speed of memory together with decreased N100 and P300 latencies. Moreover, decreased AChE was also observed.

**Figure 5:**
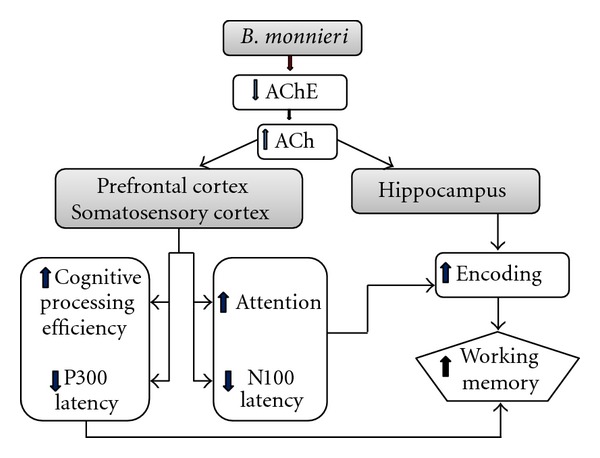
Hypothetical diagram of the possible underlying mechanism for the cognitive enhancing effect of *B. monnieri* in healthy elderly volunteers.

The assessment of selective attention, which brings targeted information into the focus of consciousness, while suppressing the detection and processing of nontarget signals, can be performed by assessing N100 component of ERP [27], whereas assessment of mental processes such as recognition, categorization of stimuli, expectancy, or short-term memory can be performed by assessing P300 component, a large positive voltage deflection in the interval between 300 and 500 ms following the stimulus onset [28]. As age advances, the latencies of P300 and N100 components increase [9, 10, 29], whereas the amplitudes of these two components are decreased [12, 29]. Changes in both parameters have been reported to be associated with decreased memory function [30–32]. A profile of evidence during the past decade has also shown that the alteration of N100, which reflects attention, and the alteration of P300, which reflects both attention and memory operation or cognitive processing, are associated with the function of cholinergic system [33, 34]. In addition, the activity of P300 has also been associated with the function of parietal cortex [30] and hippocampus [35, 36]. The current findings clearly show that subjects who consumed *B. monnieri *demonstrated enhanced attention and cognitive processing capability together with enhanced working memory and cholinergic function. The mechanism for this might be that *B. monnieri* suppresses the function of AChE in the cerebral cortex, especially in the parietal cortex and hippocampus, leading to increased available ACh in the mentioned area, giving rise to enhanced attention and memory operation capability or cognitive processing and finally resulting in enhanced working memory as shown in Figure 5. Interestingly, enhanced working memory was still presented at 4 weeks after the cessation of *B. monnieri* consumption. The possible explanation might be partly associated with changes in brain plasticity such as enhanced neuronal density in the hippocampus, an area which plays an important role in learning and memory [14], which in turn enhanced the available functional neurons in this area and resulted in the enhanced working memory. However, no changes were found in either N100 or P300 at the week 4 periods after the cessation of *B. monnieri* consumption, although both parameters were also associated with the cholinergic function. The possible explanation might partly be because the improved cholinergic function during this period might have been high enough to trigger change of event-related potential. In addition, subjects who consumed *B. monnieri* also showed higher improvement in power of attention at week 4 after the cessation of extract consumption. Although the precise underlying mechanism is still unclearly known, we suggest that it might be related to the effect of training.

In this study, we failed to show the dependent dose. This might partly be due to the following reasons; (1) the relationship between the concentration of *B. monnieri* and the interested parameters was not a simple linear relationship because *B. monnieri* might not exert its influence directly but might exert the effect via others mediators or signal molecules. (2) The extract used in this study was a crude extract which contained numerous ingredients and the increasing *B. monnieri* concentration might also have increased other ingredients which masked the effect of the active ingredient.

## 5. Conclusions

This study confirms the health benefit of *Bacopa monnieri* for the healthy elderly. In addition, no toxicity or side effects were observed throughout this experiment. It clearly demonstrated that *B. monnieri* suppresses AChE activity resulting in enhanced cholinergic function, which in turn enhances attention and memory processing and gives rise to the increased working memory. Since mild cognitive impairment (MCI) and early phase Alzheimer's disease occur due to cholinergic degeneration and oxidative stress, this plant extract may provide a benefit in terms of decreasing memory impairment in mild cognitive impairment (MCI) and early-phase Alzheimer's disease and even in attention deficit disorder. However, these avenues of research require further investigation.

## Figures and Tables

**Table 1:** Demographic data of subjects. Data are presented as mean *± *SD. *F* and *P* values were compared between groups (*n* = 20/group).

Characteristics	Placebo	300** **mg	600** **mg	*P* value
Male/Female	5/15	7/13	11/9	—
Age (years)	64.25 ± 6.80	61.85 ± 6.63	61.75 ± 5.95	0.390
Height (cm)	155.90 ± 8.14	158.15 ± 8.37	160.00 ± 8.01	0.291
Weight (kg)	57.96 ± 9.82	58.25 ± 9.38	58.10 ± 8.35	0.995
Body mass index (kg/m^2^)	23.73 ± 2.68	23.17 ± 2.31	22.61 ± 2.00	0.332
Systolic blood pressure (mmHg)	132.40 ± 12.82	133.45 ± 11.33	133.55 ± 13.55	0.952
			
Diastolic blood pressure (mmHg)	84.55 ± 4.77	85.70 ± 5.55	84.60 ± 6.89	0.779
			
Heart rate (bpm)	73.35 ± 7.98	78.90 ± 8.25	75.75 ± 9.64	0.135
Education (years)	5.05 ± 2.01	5.50 ± 3.15	5.45 ± 3.33	0.865
Full scale IQ	96.50 ± 2.48	96.30 ± 3.37	97.00 ± 4.14	0.799

**Table 2:** The effect of repetitive administration of *B. monnieri* on event-related potential. Data are presented as mean ± SD. (*n* = 20/group).

Wave	Group	Baseline	4-week	8-week	12-week	Delay
N100 latency	Placebo	103.6 ± 5.62	103.45 ± 4.58	103.15 ± 2.10	103.45 ± 4.09	103.55 ± 3.74
	300 mg	103.25 ± 3.30	101.05 ± 3.96	101.35 ± 3.36	99.65 ± 3.29***	100.35 ± 3.68
	600 mg	103.85 ± 2.9	102.05 ± 2.89	102.25 ± 2.29	100.8 ± 2.62*	102.75 ± 3.98
		*F(2, 57) = 0.03, P = 0.969 *	*F(2, 57) *= *1.26, P = 0.291 *	*F(2, 57) = 2.31, P = 0.108 *	*F(2, 57) = 6.63, P = 0.003 *	*F(2, 57) = 3.82, P = 0.028 *

N100 amplitude	Placebo	7.85 ± 3.75	7.9 ± 3.12	7.85 ± 3.67	7.7 ± 3.06	7.75 ± 4.05
	300 mg	7.65 ± 3.71	8.1 ± 3.74	8.5 ± 3.71	8.64 ± 3.15	8.5 ± 3.36
	600 mg	7.55 ± 3.69	7.73 ± 4.10	8.1 ± 3.88	8.11 ± 3.04	7.8 ± 3.56
		*F(2, 57) = 0.034, P = 0.97 *	*F(2, 57) *= *0.060, P = 0.94 *	*F(2, 57) = 0.136, P = 0.873 *	*F(2, 57) = 0.344, P = 0.711 *	*F(2, 57) = 0.261, P = 0.771 *

P300 latency	Placebo	326 ± 8.33	326.55 ± 10.01	326 ± 5.71	326.15 ± 4.63	326.00 ± 10.61
	300 mg	326.4 ± 5.68	323.8 ± 5.50	321.3 ± 5.89*	319.9 ± 4.65**	324.55 ± 6.12
	600 mg	326.2 ± 8.41	324.45 ± 8.07	321.2 ± 8.52	320.65 ± 7.30*	326.85 ± 7.47
		*F(2, 57) = 0.004, P = 0.99 *	*F(2, 57) *= *0.633, P = 0.53 *	*F(2, 57) = 3.223, P = 0.047 *	*F(2, 57) = 7.235, P = 0.002 *	*F(2, 57) = 0.394, P = 0.677 *

P300 amplitude	Placebo	10.4 ± 6.80	10.6 ± 6.51	10.55 ± 4.72	10.55 ± 6.10	10.5 ± 3.39
	300 mg	10.3 ± 4.06	11.7 ± 6.06	12.75 ± 5.91	12.75 ± 5.75	12.45 ± 6.47
	600 mg	10.45 ± 4.63	10.85 ± 5.44	11 ± 5.69	11.1 ± 4.55	10.95 ± 5.08
		*F(2, 57) = 0.004, P = 0.99 *	*F(2, 57) *= *0.183, P = 0.83 *	*F(2, 57) = 0.902, P = 0.411 *	*F(2, 57) = 0.863, P = 0.428 *	*F(2, 57) = 0.789, P = 0.459 *
